# Heart Rate Profiles During Exercise and Incident Parkinson's Disease

**DOI:** 10.1002/ana.70010

**Published:** 2025-08-02

**Authors:** Stefan van Duijvenboden, Julia Ramírez, Job Scheurink, Sirwan K. L. Darweesh, Michele Orini, Andrew Tinker, Patricia B. Munroe, Jos Thannhauser, Luc Evers, Joanna IntHout, Pier D. Lambiase, Bastiaan R. Bloem, Aiden Doherty, Marc A. Brouwer

**Affiliations:** ^1^ Nuffield Department of Population Health University of Oxford Oxford UK; ^2^ Institute of Cardiovascular Science, UCL London UK; ^3^ Clinical Pharmacology and Precision Medicine, William Harvey Research Institute, Barts and The London School of Medicine and Dentistry, Queen Mary University of London London UK; ^4^ Aragon Institute of Engineering Research, University of Zaragoza Zaragoza Spain; ^5^ Biomedical Research Networking Centre on Bioengineering, Biomaterials and Nanomedicine Madrid Spain; ^6^ Department of Cardiology Radboud University Medical Center Nijmegen the Netherlands; ^7^ Donders Institute for Brain, Cognition and Behaviour, Department of Neurology Center of Expertise for Parkinson and Movement Disorders, Radboud University Medical Center Nijmegen the Netherlands; ^8^ Barts Heart Centre, St Bartholomews Hospital London UK; ^9^ NIHR Barts Cardiovascular Biomedical Research Unit, Barts and The London School of Medicine and Dentistry, Queen Mary University of London London UK; ^10^ IQ Health, Radboud Institute for Health Sciences, Radboud University Medical Center Nijmegen the Netherlands

## Abstract

**Objective:**

To determine whether established heart rate parameters of exercise, related to cardiac autonomic function, are associated with incident Parkinson's disease, independent of both clinical and autonomic prodromal features.

**Methods:**

A study of UK Biobank participants who performed a standardized bicycle exercise test (2009–2013), followed until November 2022, and analyzed in January 2024, was carried out. Heart rate increase from rest to exercise, and heart rate decrease from peak exercise to recovery were associated with incident Parkinson's disease. Multivariable adjustment was performed both for clinical characteristics and for prodromal non‐cardiac autonomic features.

**Results:**

A total of 69,288 eligible participants (men 48%, mean age 56.8 years [SD 8.2 years]) were followed for 12.5 years: among the 319 (0.5%) who developed Parkinson's disease, recognized prodromal markers (constipation, bladder dysfunction) were more common at baseline. The median lag time to diagnosis was 9.3 years (interquartile range 4.4). Both heart rate increase (37.5 [SD 11.5] vs 40.8 [SD 12.4] b.p.m., *p* < 0.001) and recovery (23.4 [SD 8.8] vs. 27.8 [SD 10.3] b.p.m., *p* < 0.001) were significantly lower in incident cases compared with controls. Heart rate recovery was independently associated with incident Parkinson's disease, whereas heart rate increase was not. Specifically, a blunted heart rate lowering during recovery was associated with a 30% higher risk of incident Parkinson's disease (HR 1.3; 95% CI 1.1–1.4; *p* < 0.001 per 10 beats less recovery).

**Interpretation:**

Collectively, this suggests that cardiac autonomic involvement precedes clinically manifest Parkinson's disease, and that heart rate recovery might serve as a quantitative prodromal marker. ANN NEUROL 2025;98:1004–1013

Parkinson's disease (PD) is the world's fastest growing neurological disorder in terms of prevalence.^1^ One main clinical challenge lies in earlier detection, as significant neurological degeneration has already occurred by the time PD is identified with the prevailing diagnostic approaches.^2^ At present, prediction models for the early diagnosis of PD consist of predominantly non‐modifiable, binary clinical characteristics (eg, male sex or presence of hyposmia), resulting in rather “static” models.^3^, ^4^ In contrast, a continuous, more dynamic variable that may reflect quantitative changes over time during follow‐up measurements could offer additional insights. Such a biomarker could improve early diagnosis and capture the gradual progression toward manifest PD.^2^, ^5^ In this context, electrocardiographic (ECG) parameters that focus on cardiac autonomic measures have been proposed as a potentially important tool.^6^, ^7^

In patients with manifest PD, altered cardiac autonomic function is common,^8^, ^9^ including impaired heart rate regulation at rest and during exercise.^10^, ^11^ Accumulating evidence suggests that dysregulation of autonomic processes in general, and cardiac autonomic dysfunction in particular, may precede the motor symptoms of PD.^6^, ^7^, ^8^, ^9^ Importantly, pathological studies in PD have shown a characteristic sequence of neurodegenerative events, with early involvement of the dorsal motor nucleus of the vagal nerve,^12^ which regulates (cardiac) parasympathetic tone. Cardiac imaging studies have also shown changes in the sympathetic nervous system in very early stages of PD.^9^, ^13^ This background has motivated different initiatives in population‐based cohorts to search for potential prodromal autonomic markers, including heart rate regulation.^14^

Owing to differences in population characteristics, variations in choice of autonomic markers, and non‐uniformity in protocols used to assess these markers, the available evidence is difficult to interpret.^15^, ^16^, ^17^, ^18^ Two large cohort studies focused on heart rate variability (HRV), a marker of cardiac parasympathetic tone, as a potential prodromal marker for incident PD and reported conflicting findings.^15^, ^16^ Similar discrepancies in findings exist for heart rate profile studies suggesting impaired sympathetic activity as prodromal sign for PD.^17^, ^18^ Here, we focus on two markers that are recognized heart rate parameters of exercise, with established clinical value in various populations; namely, heart rate recovery and heart rate exercise.^19^, ^20^ Both these parameters of interest are related to autonomic function, and have the advantage that they are easily extractable and straightforward to assess. Heart rate recovery represents heart rate decrease from peak exercise to recovery, and is a recognized marker of parasympathetic activity^20^, ^21^, ^22^, ^23^, ^24^, ^25^ with a high reproducibility, in contrast to HRV.^26^, ^27^ Heart rate exercise represents the increase in heart rate from rest to exercise, and is primarily driven by sympathetic activation.^22^

We therefore studied these markers in a long‐term follow‐up study of 69,288 UK Biobank participants who performed an exercise test, which represents the largest investigation to date on cardiac autonomic parameters and incident PD. Our specific aim was to assess whether exercise‐related parameters of cardiac autonomic function during a baseline bicycle test would be associated with incident PD during long‐term follow up.

## Methods

### Participants

The UK Biobank study comprises a total of 502,364 participants, with even numbers of men and women aged 40–69 years at recruitment from 21 assessment centers across England, Wales, and Scotland, with extensive baseline and follow‐up clinical, biochemical, genetic, and outcome measures. The study received approval from the North West Multi‐Center Research Ethics Committee, and all participants provided informed consent at the time of enrollment (2006–2010). From the full cohort, 96,524 participants consented to participate in an exercise stress test with heart rate monitoring (2009–2013). According to protocol, 13,962 participants were considered ineligible to perform the activity (https://biobank.ndph.ox.ac.uk/ukb/ukb/docs/Cardio.pdf). Another 7,221 participants who did not complete the test were also excluded. Furthermore, exclusions were based on a history of cardiovascular disease (n = 4,441), a diagnosis of cancer within 1 year before or after the exercise stress test (n = 1,526), and a history of a neurodegenerative (n = 260) disorder (Table S1, definitions of disease/covariates). In particular, we focused on the exclusion of patients with PD at the time of the exercise test (n = 97) using previously described diagnostic criteria (https://biobank.ctsu.ox.ac.uk/crystal/ukb/docs/alg_outcome_pdp.pdf). Thus, self‐reported diagnoses were also considered, to ensure that all potential cases with PD were excluded at baseline.

**FIGURE 1:**
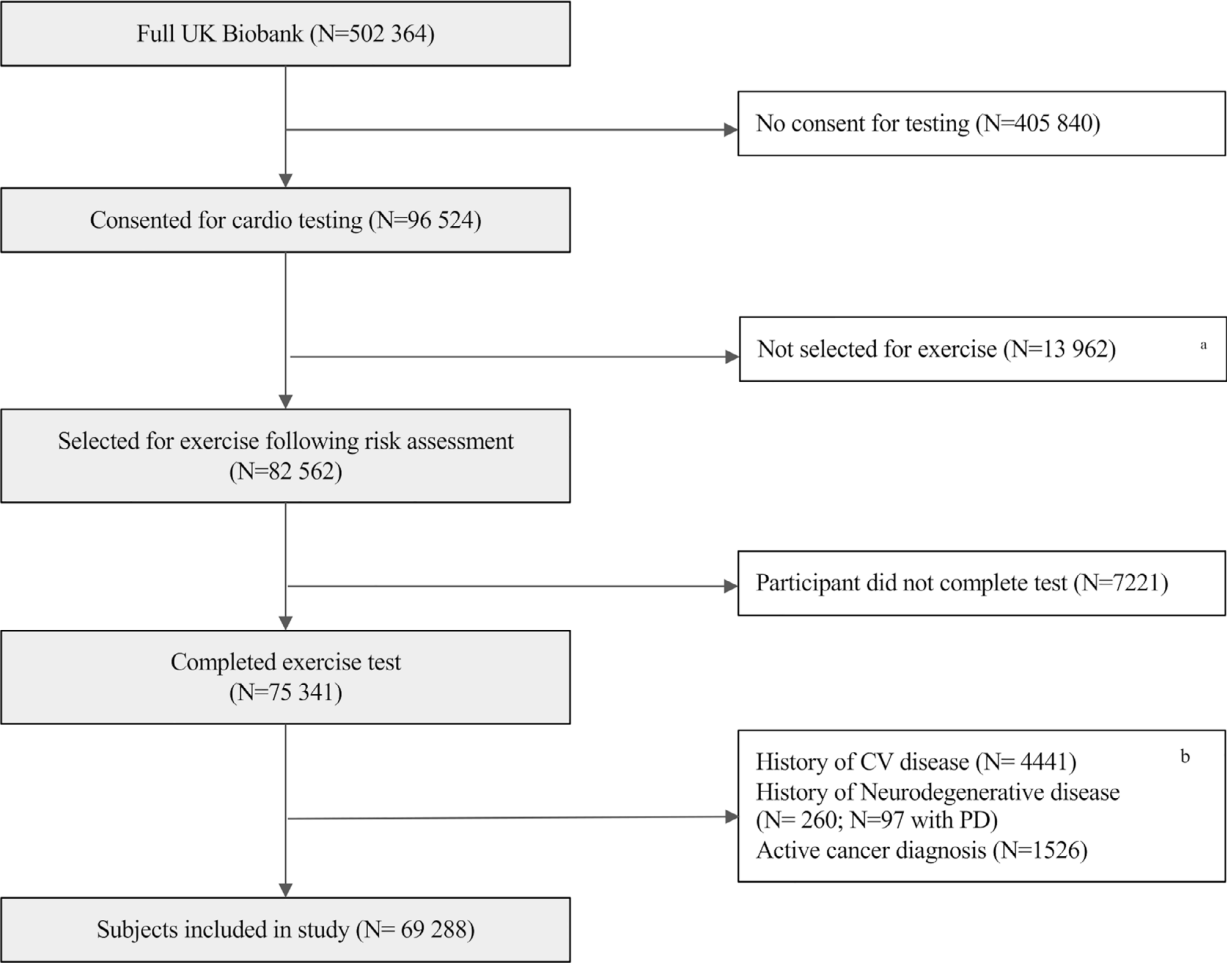
Study exclusion diagram. The full list of UK Biobank variables used for disease exclusions is provided in Table S1. ^a^Participants ineligible to exercise had 1 of more safety risk factors as defined in paragraph 9.1 of the protocol manual provided by UK Biobank (https://biobank.ndph.ox.ac.uk/showcase/ukb/docs/Cardio.pdf). ^b^Excluded participants who met one or more of the listed criteria; CV = cardiovascular; PD = Parkinson's disease.

In total, 69,288 of the 96,524 (71.8%) participants who provided consent to participate in the exercise test were included in the present analysis (Fig 1).

### Exercise Protocol

The test uses cycle ergometry on a stationary bike (eBike; Firmware v1.7) in conjunction with a single‐lead ECG device (CAM‐USB 6.5; Cardiosoft v6.51). According to protocol, the predicted absolute maximum workload was calculated, and participants were instructed to perform the test with a predefined target power at 35% or 50% of the maximum predicted workload according to the risk categories established previously (https://biobank.ndph.ox.ac.uk/ukb/ukb/docs/Cardio.pdf). In the following order, the exercise bicycle protocol consisted of a resting phase (15 s pretest), followed by graded activity with increasing workload. Per protocol, the exercise phase stopped at 6 min, after which a recovery phase followed with hands remaining on the handlebars while remaining still and silent (1 min, no cool‐down period).

### Cardiac Exercise Parameters

From the measurements recorded at 3 different phases of the exercise test we calculated: (1) “heart rate exercise”,^19^, ^22^ and (2) “heart rate recovery”^19^, ^20^, ^21^, ^22^:

1. The heart rate increase during exercise (HR‐increase): heart rate at peak exercise minus resting heart rate;
2. The heart decrease during recovery (HR‐recovery): heart rate at peak exercise minus recovery heart rate.

Heart rate parameters were calculated as follows: the ECG system computed a rolling average heart rate throughout the exercise protocol. For rest, we averaged heart rate values collected during the 15‐s pretest resting protocol. For peak heart rate, we extracted the heart rate recorded during the final ramp (~10s) of the exercise phase. For heart rate at 1‐min recovery, we extracted the heart rate recorded during the final 10 s of the recovery phase.

### Outcome Measure and Follow‐Up

Diagnoses were captured using the “Spell and Episode” category from the UK National Health Service (NHS) coded according to the 9th and 10th revisions of the International Classification of Diseases (ICD‐9 and ICD‐10), made during hospital stay, including stays from before the study inclusion. For diagnostic validity, we refer to https://biobank.ndph.ox.ac.uk/ukb/ukb/docs/alg_outcome_pdp.pdf. Detailed information about the linkage procedure is available online (https://biobank.ndph.ox.ac.uk/ukb/ukb/docs/HospitalEpisodeStatistics.pdf). The last follow‐up check was performed on November 30, 2022. Incident PD was defined as ICD‐10 code G20 (https://biobank.ndph.ox.ac.uk/ukb/ukb/docs/alg_outcome_pdp.pdf).

### Statistical Analysis

Descriptive statistics are presented as the mean (SD), median (interquartile range [IQR]), or frequencies. Continuous data were compared using Student's *t* tests or Mann–Whitney *U* tests, whichever appropriate. Categorical variables were compared using either *χ* ^2^ tests or Fisher's exact tests. The associations between exercise parameters and incident PD were investigated using 3 multivariable‐adjusted Cox proportional hazards regression models: (1) a minimally adjusted analysis using only age and sex as covariates to test whether exercise parameters were associated with incident PD; (2) a clinically adjusted model to address whether this association was confounded by demographic and clinical characteristics, other than established autonomic prodromal markers (model 1+ ethnic background, smoking, body mass index, Townsend deprivation index, and type 2 diabetes); and (3) a fully adjusted model to evaluate whether the association had incremental value beyond other autonomic prodromal markers (constipation, depression, bladder dysfunction, and self‐reported sleep duration and sleeplessness).

In previous work on heart rate recovery assessed after 1 min, the most commonly used thresholds to describe the association with all‐cause mortality were <12 b.p.m in case of passive supine recovery, and <18 b.p.m. after unloaded cycling or slow walking.^24^, ^28^ Due to a lack of comparable studies on incident PD, we decided to present the association between heart rate parameters and incident PD per unit SD.

Definitions of covariates are provided in Table S1. Schoenfeld residuals were used to test the proportional hazards assumption, and no violation was observed. Time‐to‐event curves were constructed with Kaplan–Meier methods, with log‐rank testing for statistical comparisons. Missing variables were imputed using the multiple imputation by chained equations approach, with 5 imputed datasets and 10 iterations, including comparisons of distribution plots of recorded and imputed variables.^29^ A *p* < 0·05 was considered statistically significant. Statistical analyses were performed in R v4.2.0 using the survival (v3.5.8; The R Foundation for Statistical Computing, Vienna, Austria) and mice (v3.15.0; The R Foundation for Statistical Computing) libraries.^30^

### Sensitivity Analyses

We prespecified sensitivity analyses to assess whether the associations found in the main analysis were affected by: (1) heart rate modulating agents (ie, beta and calcium blockers); and (2) psychoactive drugs (excluding, for example, participants exposed to drugs affecting the dopaminergic system). Also, we performed analyses with and without inclusion of participants for whom data were imputed. Post‐hoc sensitivity analyses were performed to further explore the associations observed in the prespecified analyses.

## Results

In total, there were 96,524 participants who consented to partake in the exercise test, of whom 27,236 were excluded (Fig 1, Table S2). Excluded participants showed clinically relevant differences in baseline demographics (older age and body mass index) and in established prodromal factors of incident PD (higher prevalence of risk factors) compared with the remaining 69,288 individuals (Table S3).

Incident PD was diagnosed in 319 individuals (0.5%; Table 1). At the time of the exercise test, participants who developed PD during follow up were older than those who remained disease free and were more often male. Rates of many of the prodromal risk factors (such as constipation) were higher, whereas the rate of smoking (protective factor) was lower.

**TABLE 1:** Baseline Characteristics of Participants With and Without Diagnosis of Incident Parkinson's Disease During Long‐Term Follow‐Up

	PD (*n* = 319; 0.5%)	No PD (*n* = 68,969; 99.5%)	*n*	*p* ^a^
Sex, male, n (%)	221 (69.3)	32,732 (47.5)	69,288	<0.001
Age, years, mean (SD)	63.2 (5.2)	56.8 (8.2)	69,288	<0.001
Ethnicity, n (%)			68,905	0.089
White	303 (95.3)	63,868 (93.1)		
Black	5 (1.6)	1,540 (2.2)		
Asian	10 (3.1)	1,552 (2.3)		
Chinese	0 (0.0)	282 (0.4)		
Mixed	0 (0.0)	562 (0.8)		
Other	0 (0.0)	783 (1.1)		
BMI, mean (SD)	27.6 (4.0)	27.0 (4.4)	69,287	0.001
Townsend deprivation index^a^, mean (SD)	−1.6 (2.8)	−1.4 (2.9)	69,215	0.079
Type 2 diabetes, yes, n (%)	42 (13.2)	2,889 (4.2)	69,288	<0.001
Current smoking, yes, n (%)	10 (3.2)	5,616 (8.2)	68,977	0.001
Constipation, yes, n (%)	19 (6.0)	2,084 (3.0)	69,288	0.002
Depression, yes, n (%)	28 (8.8)	3,776 (5.5)	69,288	0.010
Bladder dysfunction, n (%)	6 (1.9)	820 (1.2)	69,288	0.29
Erectile dysfunction, n (%)	5 (2.3)	143 (0.4)	32,953	0.003
Sleep duration, h, mean (SD)	7.3 (1.3)	7.1 (1.1)	69,182	0.024
Sleeplessness, n (%)	73 (23.1)	17,428 (25.4)	69,035	0.36

^a^ Townsend deprivation index is a measure of material deprivation.

BMI = body mass index; PD = Parkinson's disease.

Median follow‐up duration on survival status was 12·5 years (IQR 0·3) with >10 years of follow up available in 59,460 participants (85.6%). During this follow‐up period, 3,106 (4.5%) participants died.

### Heart Rate Response to Recovery is Blunted in Prodromal PD

During the pretest stage, resting heart rate was not significantly different between participants who developed PD and those who did not (Table 2). However, the increase in heart rate during exercise (HR‐increase) and decrease during recovery (HR‐recovery) were both lower in the group that developed PD with small‐to‐moderate effect sizes (Cohen's *d* = 0.27 and 0.43, respectively; Table 2).

**TABLE 2:** Heart‐Rate Markers During Exercise Testing Comparing Participants With and Without Incident PD During Long‐Term Follow‐Up

	PD (*n* = 319; 0.5%)	No PD (*n* = 68,969; 99.5%)	*n*	Cohen's *d*	*p*
Resting heart rate, mean (SD)	70.6 (11.5)	70.2 (11.1)	68,103	0.036	0.86
HR‐increase, mean (SD)	37.5 (11.5)	40.8 (12.4)	67,553	0.267	<0.001
Peak heart rate, mean (SD)	107.9 (14.1)	111.0 (13.9)	67,780	0.222	<0.001
HR‐recovery, mean (SD)	23.4 (8.8)	27.8 (10.3)	67,109	0.427	<0.001
Heart rate at recovery, mean (SD)	84.7 (14.5)	83.3 (14.0)	67,524	0.105	0.053

HR‐increase = heart rate increase during exercise phase (ie, heart rate at peak exercise minus resting heart rate); HR‐recovery = heart rate decrease during recovery phase (ie, heart rate at peak exercise minus heart rate at recovery); PD = Parkinson's disease.

The results of the univariate Cox regression analysis are presented in (Table S4). The established prodromal clinical variables were significantly associated with incident PD. With regard to cardiac autonomic parameters, HR‐increase was associated with a 30% higher risk of incident PD per 10 beats less increase in heart rate during exercise. For HR‐recovery, a blunted lowering in heart rate from peak exercise to recovery was associated with a 60% higher risk of incident PD per 10 beats difference.

In the minimally adjusted Cox regression analysis, HR‐recovery was significantly associated with incident PD, and HR‐increase was not (Table 3). After adjusting for clinical and autonomic prodromal factors, it was found that a blunted lowering in heart rate during recovery remained independently associated with a 30% higher risk of incident PD: HR 1.3; 95% CI 1.1–1.4; *p* < 0·001 per 10 beats less recovery (Tables 3 and S5).

**TABLE 3:** Association of Exercise Heart‐Rate Markers With Incident Parkinson's Disease, Sequential Adjustment for Clinical and Autonomic Factors

Model	Marker	HR per 10 b.p.m. (95% CI)	*p* value
Adjusted for age and sex			
	HR‐increase	1.0 (0.9–1.1)	0.48
	HR‐recovery	1.3 (1.2–1.5)	<0.001
Adjusted for clinical variables^a^			
	HR‐recovery	1.3 (1.1–1.5)	<0.001
Adjusted for clinical and prodromal autonomic variables^b^, ^c^			
	HR‐recovery	1.3 (1.1–1.4)	<0.001

Hazard ratios are expressed per 10 b.p.m. impairment.

^a^ Clinical variables: age, sex, ethnicity, body mass index, Townsend deprivation index, type 2 diabetes, and smoking.

^b^ Prodromal autonomic variables: constipation, sleep duration, sleeplessness, depression, and bladder dysfunction.

^c^ The interaction term between heart rate at peak‐exercise and HR‐recovery was not statistically significant (*p* = 0.53).

HR‐increase = heart rate increase during exercise; HR‐recovery = heart rate decrease during recovery.

**FIGURE 2:**
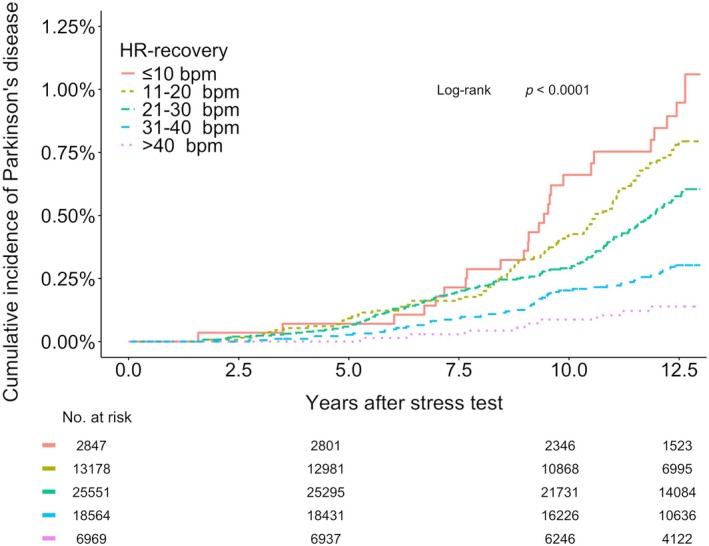
Unadjusted Kaplan–Meier estimates stratified by heart rate decrease during recovery (HR‐recovery). [Color figure can be viewed at www.annalsofneurology.org]

**FIGURE 3:**
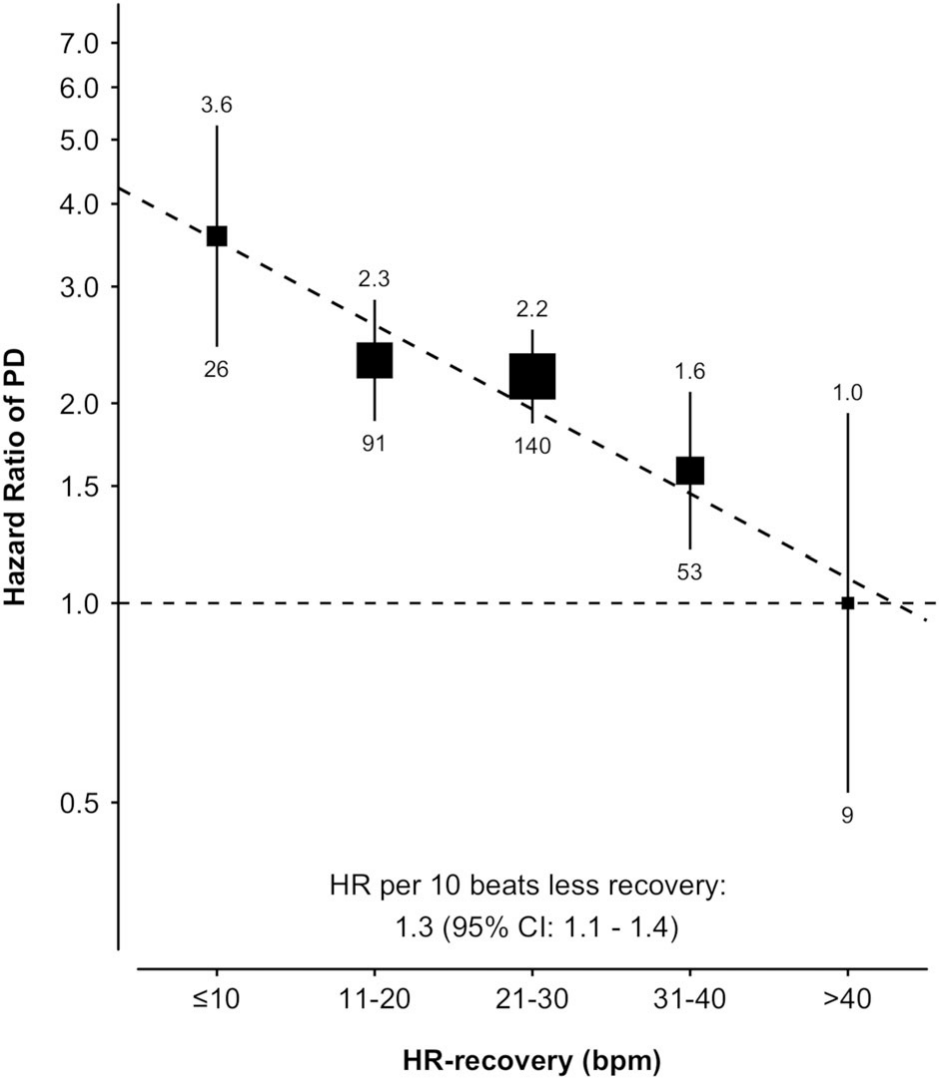
Association of heart rate decrease during recovery (HR‐recovery) with risk of Parkinson's disease. Adjusted for sex, age, ethnicity, Townsend deprivation index, body mass index, type 2 diabetes, smoking status, bladder dysfunction, constipation, depression, and self‐reported sleep duration and sleeplessness. The number above each vertical line is the hazard ratio, and the number below is the number of incident cases.

Figure 2 shows time‐to‐event curves. The median lag time to (hospital) diagnosis of incident PD was 9.3 years (IQR 4.4 years.), showing the highest risk of incident PD in the participants with the lowest HR‐recovery. The absolute difference in outcome at 12 years between participants with lowest (≤10 b.p.m.) and highest (>40 b.p.m.) HR‐recovery was 0.7% (95% CI 0.4%–1.1%). After adjusting for clinical and autonomic prodromal factors, the association between HR‐recovery and incident PD was found to be approximately log‐linear (Fig 3).

#### Sensitivity Analyses

In both prespecified sensitivity analyses with focus on medication, the association between HR‐recovery and incident PD remained significant, with similar risk estimates as the main analysis. In addition, findings and conclusions did not alter after the exclusion of individuals with imputed data (Table S6).

Post‐hoc sensitivity analyses focused on the association in relation to the achieved workload during the exercise test, and addressed the impact of imposing a longer lag time to diagnosis. Outcomes did not alter the overall conclusions of the main analysis. Finally, we studied the association in the subset of participants with both out‐of‐hospital and in‐hospital follow‐up information, and the findings were in line with the main analysis (Table S7).

## Discussion

The present study represents the largest population‐based cohort to date on the association of recognized heart rate parameters of exercise, known as indicators of autonomic function, with the highest number of incident PD cases (*n* = 319) reported in this setting. An impaired capacity to reduce the heart rate during recovery immediately after exercise was associated with incident PD, independently of established prodromal clinical and autonomic markers. Notably, our findings do not indicate a threshold value, but rather, suggest a more graded association between a blunted heart rate recovery after exercise and incident PD (Figs 2 and 3). There was a univariate association between heart rate increase during the exercise phase and incident PD, which was no longer present after adjustment for sex and age. We also replicated earlier work by showing that individuals with prodromal parkinsonism had a greater likelihood of having established prodromal markers at baseline, including indicators of non‐cardiac autonomic dysfunction (ie, constipation etc.). This suggests that this study cohort was representative of previously studied prodromal populations. Analogous to established prodromal markers,^31^ there was a median lag time to diagnosis of approximately 9 years. This represents the time until hospital diagnosis, thus the actual lag time to an outpatient diagnosis is shorter. In terms of future perspectives, we now present a candidate marker that is continuous, whereas most prodromal markers are “static” binary variables (eg, sex, hyposmia). Therefore, this marker has potential to be “dynamic,” as it may reflect quantitative changes over time by use of follow‐up measurements.^32^, ^33^ Longitudinal studies are warranted. As a quantifiable, continuous parameter, this cardiac autonomic marker may not only offer early diagnostic value as part of a battery of other markers,^4^, ^9^, ^34^ but also has promise to capture more subtle progressive changes during the prodromal phase. If proven to be modifiable in PD, it might be relevant when used as an outcome measure in studies of putative disease‐modifying interventions applied in the prodromal phase.^2^, ^14^, ^35^

Immediately after cessation of exercise, the key physiological response is a parasympathetic reactivation, leading to a quick decline in heart rate in the first minute.^20^, ^21^, ^22^, ^23^, ^24^, ^25^ The blunted heart lowering observed in PD cases may therefore be suggestive of parasympathetic autonomic dysfunction, in line with Braak's staged disease development, with involvement of the dorsal nucleus of the vagal nerve as one of the early manifestations that may antedate clinically manifest motor PD.^8^, ^12^, ^13^, ^36^, ^37^ This is further supported by the observation of the marked differences in other autonomic prodromal markers at the time of the exercise test (eg, constipation, bladder dysfunction) between participants who developed PD during follow‐up versus those who did not. Importantly, heart rate recovery is also a recognized indicator of cardiovascular fitness,^38^ which is why we included a sensitivity analysis in relation to workload.

Regardless of workload, the association between heart rate recovery and incident PD was similar in direction and magnitude, which further strengthens our conclusions (Table S7).

With regard to previous work on parasympathetic involvement, the Atherosclerosis Risk in Community cohort studied HRV at rest and found an association with incident PD. During 20 years of follow up in 12,162 patients (57% women, mean age 54 years), PD was diagnosed in 0.6% (78 cases).^15^ The Cardiovascular Health Study (*n* = 5,888) studied a community‐based population (58% women, mean age 73 years), and HRV was assessed using 24‐h Holter monitoring.^16^ During 14 years of follow up, PD was diagnosed in 3%, and in the subset (*n* = 1,587) with Holter monitoring no association was found between HRV and incident PD.^16^ The interpretation of these discrepant findings is difficult, not only because of the non‐uniformity between studies in the assessment of HRV (in‐ or exclusion of activity; ECG recordings of 2 min vs 24 h). Also, HRV as a marker of parasympathetic function^26^ is susceptible to reproducibility issues, as it is strongly dependent on other physiological factors.^26^, ^27^

With regard to heart rate exercise, primarily driven by sympathetic activation,^19^, ^22^ we observed that the heart rate increase during exercise was lower for individuals who developed PD during follow up, but the association was no longer present after adjustment for sex and age. Both in manifest motor PD, and in individuals in the prodromal phase, sympathetic involvement has been demonstrated.^8^, ^9^, ^13^, ^36^, ^39^ Although a small case–control study on heart rate profiles during maximal exercise suggested sympathetic dysfunction during the premotor phase of PD,^17^ this could not be confirmed in a much larger study.^18^ Explanations for the discrepant findings may lie in methodological aspects, such as maximal versus submaximal exercise,^40^ differences in sample size, or the selection of the incorporated confounding variables.

Our study is not without shortcomings. First, we acknowledge limitations in terms of generalizability with this rather healthy cohort. Yet, with this particular study population, the association between heart rate recovery and incident PD is less likely to be flawed by comorbidity. Importantly, the heart parameters of interest are indicators of autonomic function, but many other aspects may influence the responses to exercise and subsequent cessation.^41^ Factors, such as use of coffee or the intensity of exercise the day before/of the test, were not accounted for. The strict exclusion criteria applied here, the many corroborative sensitivity analyses (including medication used) and the finding that non‐cardiac prodromal markers of autonomic dysfunction also markedly differed between groups support the notion that the difference in heart rate recovery is likely to reflect parasympathetic involvement. Second, the present work is a post‐hoc analysis. As incident PD was not the primary outcome of interest, we applied a strict case definition supported by source documents (a hospital admission confirming the ICD‐code for PD). This may have caused an underestimation of the actual incidence and may have influenced the effect estimations. Moreover, it may result in a different case load, as patients with a hospital diagnosis may vary in clinical profile from those with an out‐of‐hospital diagnosis. Notably, the hospital‐based diagnosis was systematically collected during follow up, in contrast to out‐of‐hospital diagnoses. A post‐hoc sensitivity analysis using an alternative definition for incident PD; that is, new out‐of‐hospital and/or in‐hospital diagnosis of PD (Table S7), resulted in outcomes that were in line with the overall conclusions of the main analysis. With regard to the clinical profile of the cases in this sensitivity analysis, we observed that approximately 30% had an earlier out‐of‐hospital diagnosis, diagnosed 2.4 years earlier than the in‐hospital diagnosis. Appreciating that the majority of in‐hospital diagnoses were first diagnoses, an interesting hypothesis is that these may have been more severe cases, as early involvement of body structures outside the nervous system (including the gut and the heart) is associated with a more malignant phenotype and a more rapid disease progression.^13^

We also acknowledge that the differential diagnosis from atypical parkinsonism (where the nature and extent of autonomic dysfunction is different) can be challenging, and a misclassification rate of up to 15% occurs in clinical studies of PD patients. However, such a misclassification would not have markedly affected the associations that we found. Finally, our registrations did not meet the required duration and physical conditions to assess HRV,^26^ which precludes comparisons with the previous cohorts.^15^, ^16^

We foresee several areas for further research. First, our findings remain to be confirmed; then, initiatives to define a diagnostic cutoff value can be substantiated. A combination of a parasympathetic heart rate parameter and imaging markers of cardiac denervation^37^, ^42^ could be a future perspective. Notably, in a well‐defined population with an a priori higher risk, the diagnostic value of imaging evidence of cardiac denervation seems promising.^42^ Second, follow‐up studies measuring changes of heart rate markers^32^, ^33^ may provide insight into the potential to capture progression of disease. Third, heart rate recovery is a modifiable parameter; for example, by training^43^, ^44^ and drugs.^23^, ^24^, ^25^ Whether this modifiability also applies to a prodromal population in various stages of parasympathetic involvement and/or cardiac denervation remains to be determined. Potentially, a therapeutic intervention that effectively slows disease progression may also affect heart rate recovery. In that case, heart rate recovery could become a quantitative indicator of therapeutic interventions. Interestingly, training has shown positive effects in PD patients, but the underlying mechanism and whether this can be monitored by heart rate recovery is subject of further study.^45^

In general, future studies should focus on uniform methodology,^26^, ^27^ as this improves the scientific quality of pooled analyses, required to more thoroughly study infrequent diseases. Moreover, detailed descriptions of study participants are warranted, with the growing notion of distinct patterns in the development toward PD, defining a brain‐first and a body‐first subtype.^13^ Studies in these subpopulations^2^, ^14^ are warranted. For example, the potential diagnostic value of heart rate recovery may be greater in the body‐first subtype, where autonomic involvement occurs much earlier in the disease process.

Taken together, our findings provide additional supportive evidence for the concept that pathophysiological changes in the autonomic nervous system may precede the clinically manifest motor syndrome of PD. If confirmed in an independent cohort, we anticipate that autonomic cardiac biomarkers could be incorporated in a more comprehensive battery of early diagnostic biomarkers, varying from clinical characteristics, autonomic markers, and imaging and tissue markers.^4^, ^33^, ^40^

## Author Contributions

S.vD., M.B., B.B., and A.D. contributed to the conception and design of the study, S.vD., M.B., J.R., M.O., J.I., B.B., and A.D. contributed to the acquisition and analysis of data; S.vD., M.B., J.R. J.S. S.D, M.O., A.T., P.M. J.T., L.E., J.I., P.L., B.B, and A.D. contributed to drafting the text and preparing the figures.

## Potential Conflicts of Interest

Nothing to report.

## Supporting information


**Data S1.** Tables Information.

## Data Availability

Data from the UK Biobank are available to researchers on application to the UK Biobank (https://www.ukbiobank.ac.uk/enable-your-research/about-our-data).
